# Structure of nods in conversation

**DOI:** 10.1371/journal.pone.0323448

**Published:** 2025-05-22

**Authors:** Taiga Mori, Yasuharu Den, Kristiina Jokinen

**Affiliations:** 1 Artificial Intelligence Research Center, National Institute of Advanced Industrial Science and Technology, Tokyo, Japan; 2 Graduate School of Science and Engineering, Chiba University, Chiba, Japan; 3 Graduate School of Humanities, Chiba University, Chiba, Japan; Università del Salento: Universita del Salento, ITALY

## Abstract

Head nods are a commonly observed gesture in daily conversations and has attracted the interest of many researchers in human, social, and computer sciences since early studies. However, there has been little research focusing on the structure of nod movement, which sometimes involve repetition of upward and downward movements. In this study, we shed light on this structure of nods which has been previously overlooked. Prior to the analysis, we propose systematic conceptualization of nod movement. First, we define a cycle as a consecutive upward and downward movement as the basic unit of analysis. We next define the number of cycles within a nod as its length and the relative position of a cycle within a nod as its position. We then define the magnitude as the difference between the lowest and highest points of the head within a cycle. In the analysis, we demonstrate that this magnitude varies depending on length and position, thereby providing evidence that nods exhibit a structured pattern. Specifically, three structural patterns were observed: (1) the magnitude of the first cycles increases with length (anticipatory rising), (2) the magnitude decreases proportionally with position (declination), and (3) the magnitude of the final cycles is noticeably smaller than predicted from the preceding trend (final lowering). Finally, we discuss the similarities between the discovered structure and the phonological structure of utterances, suggesting that these structures may represent a universal characteristic of human repetitive actions.

## Introduction

Head nods are a common gesture observed in many cultures and frequently occur during conversations. Nods have attracted considerable researchers’ attention in human, social, and computer sciences since early studies. The frequency, timing, and co-occurrence with speech have been extensively studied [1–4]. The cultural difference has also been studied [5,6]. Because of the importance of nods in conversation, recent research in artificial intelligence has focused on incorporating nods into dialogue systems. These studies primarily concentrate on techniques to detect user nods [7–10] and predict appropriate timing for the system to produce nods [11–13].

There have been numerous studies on the timing and function of nods, but very few have focused on its structure. Unlike hand gestures, which can convey high-dimensional information through the shape and movement of individual fingers and the entire arm, as well as their combinations using both hands, nods are limited to only two dimensions: vertical movement and time. As a result, the nature of nods as a movement is often taken for granted, used without a clear definition (e.g., [11]), or simply defined as “vertical up-and-down movement of the head rhythmically raised and lowered” (e.g., [7]). Consequently, the question of whether nods are merely a series of up-and-down head movements, or they possess any distinct structure has not been thoroughly investigated. To better understand how we produce nods and how they are used as an interactional resource, and to develop dialogue systems capable of generating natural nods, it is important to more deeply study the structure of nods.

This study focuses on the structure of nods which has been previously overlooked. Prior to the analysis, we propose systematic conceptualization of nod movement, which sometimes involve repetition of upward and downward movements. First, we define a cycle as a consecutive upward and downward movement as the basic unit of analysis. We next define the number of cycles within a nod as its length and the relative position of a cycle within a nod as its position. We then define the magnitude as the difference between the lowest and highest points of the head within a cycle. In the analysis, we demonstrate that this magnitude varies depending on length and position, thereby providing evidence that nods exhibit a structured pattern. We construct a statistical model that best represents this structured pattern. Finally, we discuss the similarities between the discovered structure and the phonological structure of utterances, suggesting that these structures may represent a universal characteristic of human repetitive actions.

The structure of this paper is as follows. Chapter 2 provides an overview of related research, discussing how the structure of nods has been overlooked. Chapter 3 describes the data and terminology used in this study and outlines the method for calculating the magnitude of movements composing a nod. Chapter 4 conducts statistical analysis, demonstrating some patterns in the magnitude of movements in nods. Chapter 5 discusses the structure of nods based on the results and finally, Chapter 6 presents the conclusions.

## Related research

Even though there are some annotation schemes of gestures including nods, they do not provide explicit definition of nods. Kousidis et al. [14] examined annotation schemes for head gestures and defined nods as “rotation down-up” of the head, where even a single down or up movement is considered a nod. Additionally, if there is a perceptible gap in a continuous head movement, each segment is regarded as a component of a nod, but there is no discussion about whether they compose a single or repetitive nod (cf. Bauer et al. [15], regarding the gap for segmenting nods). In the MUMIN scheme by Allwood et al. [16], nods are defined as a “head movement down-up,” and movements starting with an upward motion are classified as jerks. In their scheme, nods are categorized as single nods and repeated nods based on the frequency of head movements, although the criteria for distinguishing between single and repeated are not explicitly defined.

There are a few studies that kinetically divide nods into individual movements. Boholm and Allwood [17] segmented nods into individual up or down movements to analyze their relationship with gesture functions. In their study, a nod is defined as containing at least one upward and one downward movement, with each pair of movements counted as one nod. Their data of Swedish conversations revealed that two-time nods (two pairs of up-and-down movements) were most common, with nods starting with upward movements more frequent than those starting with downward movements. Furthermore, they noticed that Swedish notification marker “*okej* (ok)” is likely to co-occur with repeated up-nods. Likewise, some research categorizes nods based on the direction of the initial movement. Navarretta et al. [6], following the MUMIN scheme, classified nods into down-nods and up-nods (jerks) based on the initial movement direction and further categorized them as single nods or repeated nods based on the number of up-and-down movements. Their analysis across Danish, Finnish, and Swedish found that down-nods were more frequent or equally frequent compared to up-nods in all languages, with single nods being more frequent than repeated nods only in Finnish. Toivio and Jokinen [18] investigated the frequency of down-nods and up-nods, as well as the frequency of verbal responses and their co-occurrence, in Finnish conversations during the first and second encounters. They found that down-nods occur more frequently than up-nods, and that the frequency of both types of nods decreases in second encounters. Additionally, their observations suggest that down-nods and up-nods may play different roles in building shared understanding. Mori et al. [19], analyzing Japanese conversations, divided listener nods into up-nods and down-nods and analyzed the functional differences between them. The analysis revealed that down-nods are used as acknowledgements of known information, while up-nods are used as responses to new information or initiation of repairs.

Apart from descriptive studies on the form of nods, i.e., up- vs. down-nods and single vs. repeated nods, there is very little understanding of how the movement of nods changes over time. One of the few studies that mentions this aspect is the research conducted by Harder and colleagues. Harder et al. [2] recorded head movements in conversation using a polarized light goniometer and analyzed the synchronization with speech. Regarding the shape of nods, they observed that the amplitude of nods starts wide and then gradually gets narrowed, but no further analysis was conducted about it.

One hint to understanding the structure of nods may come from an influential study on gesture structure by Kendon [20]. Kendon’s gesture phase framework [20] is a widely accepted conceptual model for structuring gestures in general. According to his framework, gestural movements can be temporally divided into up to four phases: preparation, stroke, hold, and recovery. Puupponen et al. [21] proposed that the typical pattern of nod movement in Finnish Sign Language consists of three phases: preparation, stroke, and recovery. In the preparation phase, the head position shifts slightly in preparation for the stroke. The stroke phase follows, during which the chin moves toward the chest, producing the greatest amplitude. Finally, in the recovery phase, the head movement diminishes and returns to a neutral position or transitions into the next head movement. However, this structuring was intended solely to distinguish between single and repeated nods and was not the primary focus of the study. Changes in stroke amplitude depending on the number of repetitions, or the temporal variation of stroke amplitude in repeated nods, were not examined deeply.

In sum, the studies by Boholm and Allwood [17], Navarretta et al. [6], Toivio and Jokinen [18] and Mori et al. [19] are related to the structure of nods, particularly in how the initial movement of the nod is associated with the function of the nod. However, their research does not address subsequent movements or the relationships between individual movements. The studies by Puupponen et al. [21] and Harder et al. [2] suggest that there is a temporal structure in nods, but this was not the main focus of their research and no detailed analysis was conducted. Therefore, it is still unclear whether nods are merely a random up-and-down motion of the head or if it follows a distinct structural pattern. This study focuses on the magnitude of the nod motion and investigates how the magnitude changes over time and with the number of repetitions.

## Methods

### Data

**Fig 1:**
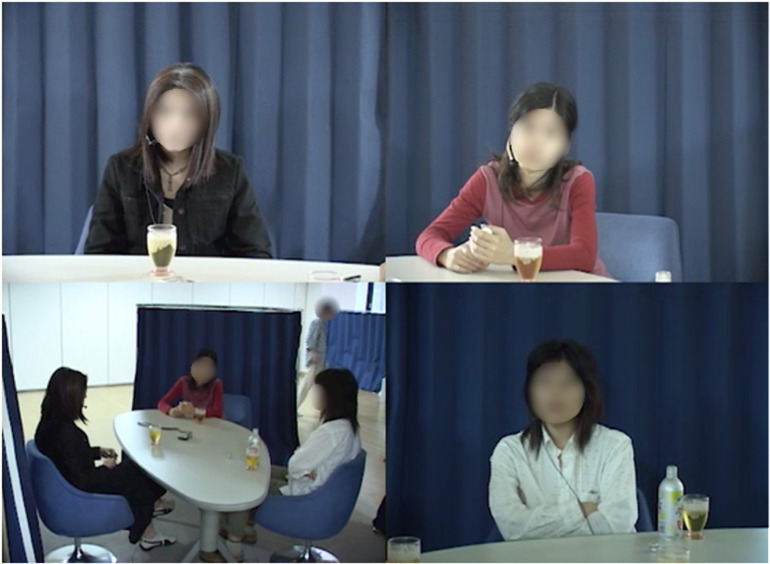
Screenshot of the corpus.

We utilized an existing conversational corpus called *the Chiba Three Party Conversation Corpus* [22] for our data (available at https://doi.org/10.32130/src.Chiba3Party). This study was conducted within the scope of this corpus’ terms and did not involve collecting new behavioral recordings. Therefore, this study itself did not undergo a new ethics review. Fig 1 provides a screenshot of the corpus. This dataset includes face-to-face casual chats among friends of university students, graduate students, and postdoctoral researchers, with a total of 36 participants divided into 12 groups, each engaging in three conversational sessions. Only the data for the second session is published, but we were allowed to use the data for the other sessions. Each session lasted 9 minutes and 30 seconds, culminating in a total duration of 342 minutes (36 conversations). Conversation topics were randomly determined using rolls of a dice whose faces show different pre-determined topics, but participants generally talked freely regardless of the selected topic. The interactions were recorded from four perspectives: front-facing videos of individual participants and an overhead-view video. Audio was captured from headset microphones worn by individual participants.

### Annotation

The corpus includes transcriptions and annotations of several sorts, including head gestures. In head gesture annotation, each gesture produced by participants is manually labeled with one of four categories: nod, shake, tilt, or other, along with segment information indicating the start and end times. However, the annotations are only available for the second session (12 conversations, 36 participants), which are officially published. In this study, we built a gesture detection model using the existing annotations and combined it with manual corrections to annotate head gestures in sessions 1 and 3 as well.

For the automatic annotation, head pose data was, first, estimated using OpenFace’s FeatureExtraction tool [23]. OpenFace estimates the 3D coordinates of facial landmarks from input images using the Convolutional Experts Constrained Local Model and projects them onto the image to estimate head pose relative to the image. Because all settings were kept at their default values, Dlib [24] was used for face detection.

Next, we built head gesture detection models based on the annotations from session 2. There are four types of models: the nod detection model, the shake detection model, and the tilt detection model, each trained with the respective annotations, and a general head gesture detection model trained with annotations of all head gestures. First, the general gesture detection model predicts the probability of a gesture occurring in each frame. This probability is then smoothed, and frames with values above the threshold of 0.5 are identified as gesture-occurring segments. Next, specific gesture prediction models estimate the probabilities of each gesture within these detected segments. Finally, the gesture with the highest probability within each segment is assigned as the gesture for that segment.

All models took the second-order differences of five frames of 3D head poses (pose_Tx, pose_Ty, pose_Tz, pose_Rx, pose_Ry, pose_Rz) estimated by OpenFace as input and predicted the presence of gestures in those frames. For the performance evaluation using the session 2 data, which contains manually annotated correct data, data from 30 individuals were used as training data, data from 3 individuals were used as validation data, and data from 3 individuals were used as test data.

The model structures were determined empirically: the head gesture detection model consisted of one fully connected layer and one bidirectional GRU layer, the nod and shake detection models each consisted of one fully connected layer and one bidirectional LSTM layer, and the tilt detection model consisted of one bidirectional LSTM layer. To limit the annotation intervals and reduce manual annotation costs, the models were trained to maximize recall on the validation data. Training was conducted over 1000 epochs, and the model with the best recall was selected. The recall of the best model on the test data was 90% for the general head gesture detection model, nod detection model, and shake detection model, and 66% for the tilt detection model.

Finally, the annotations for sessions 1 and 3 data were automatically created using these models and then manually corrected. Since the models were trained to maximize recall, they likely produced a low rate of false negatives but a higher rate of false positives. Therefore, annotators were instructed to review only the intervals where the models detected gestures, delete the label if no gesture was present there, correct the label if its type was incorrect, and adjust the interval if it was inaccurate. This procedure theoretically increased the precision to 100%, resulting in a final F1 score close to 100% as well. The final number of nods obtained was 9,230.

### Terminology

**Fig 2:**
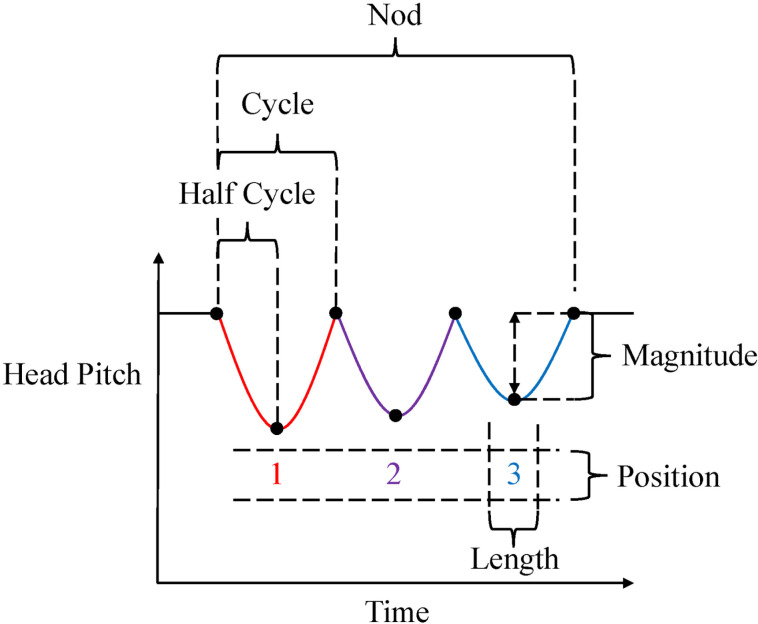
Terminology used in this study.

This study focuses on the structure of nods, which has not been previously addressed. We first propose systematic conceptualization related to nods as a head movement as shown in Fig 2.

Nods generally consist of variations in pitch (vertical rotation around the lateral axis), in other words up-and-down movements of the head. Similarly to previous studies such as [3], this research defines a *nod* as a gesture consisting of continuous one or more vertical head movements regardless of whether the nod motion begins with an upward or downward movement. Therefore, our nod includes both of down-nods and up-nods defined in [6]. A nod begins when the head starts moving from a stationary position and ends when the head comes to a stop again.

Theoretically, the smallest nod comprises a single upward or downward movement. However, often after a downward/upward movement, there is a subsequent upward/downward movement [25] to come back to the initial position. According to Kendon’s [20] framework of gesture phases, the preceding downward/upward movement and the subsequent upward/downward movement correspond to the stroke and recovery, or preparation and stroke, of a head gesture. In fact, a preliminary analysis of our corpus indicated that the first and the second movements are nearly identical in magnitude. Therefore, in practice, a pair of consecutive upward and downward movements serves as the basic unit of a nod. Since we focus on the magnitude of movements during nods in this study, we define this pair of two movements in alternate directions as the basic unit of nod and call it as a *cycle*, irrespective of the order of upward and downward movements. Likewise, we call a single upward or downward movement within a cycle as a *half-cycle*. When a nod comprises *N* half-cycles, we refer to the pair of the *i*-th and *i* + 1-th movements as one cycle (where *i* is an odd number). Sometimes nods may occasionally comprise an odd number of half-cycles [17]. In such cases, following Kousidis et al. [14], we regard the last half-cycle as a cycle.

Next, we formally define the *magnitude* of a cycle as the scalar value of the difference between the lowest and highest points of the head within a cycle. This magnitude differs from the strict definition of amplitude. Since amplitude is the distance from the center of a wave to its peak, the magnitude would be twice the amplitude if the nod motion is considered, e.g., a cosine wave. The unit of magnitude in this study is measured by “degrees.”

Finally, the total number of cycles included in the nod is referred to as *length*. This is equivalent to the number of times the participant moves the head within a nod. Therefore, *single nods* refer to nods of length 1, while *repetitive nods* refer to nods of length 2 or more. Additionally, the relative position of cycles within a nod is referred to as *position*. For example, a nod with a length of 2 consists of two cycles, where the position of the first cycles is 1, and the position of the second cycle is 2. This study focuses on how magnitude changes depending on length and position.

### Calculation of Magnitude

First, the head pitch was extracted from the OpenFace data used for annotation. Regarding the accuracy of OpenFace, according to [23], the error in head pose estimation is minimal, with an average of 2.6 degrees for the BU dataset [26] and 3.2 degrees for the iCCR dataset [27]. These datasets contain extreme head movements that are not typical of conversational scenarios (for example, in Fig 6, frame 478 of [27]). Therefore, it is expected that the error in our data would be much smaller. Additionally, our data includes 9,230 head nods, which makes it robust to error. Furthermore, as is describe below, in the estimation of inflection points, the mean absolute error between the number of half-cycles estimated by OpenFace for each head nod and those manually annotated is less than 1. From these facts, it can be concluded that OpenFace has sufficient accuracy for the purposes of this study.

OpenFace outputs pose_Rx, pose_Ry, and pose_Rz for head rotation. Among these, pitch corresponds to pose_Rx. Note that in the original output, the pitch value increases when the head moves downward, which is counterintuitive when visualized in a graph as an upward movement. Therefore, for subsequent analyses, we inverted the sign of the pitch to align the apparent motion in real world with the motion depicted in the figures. Additionally, while the original output of pose_Rx is in radians, this study converts it to degrees for clarity.

**Fig 3:**
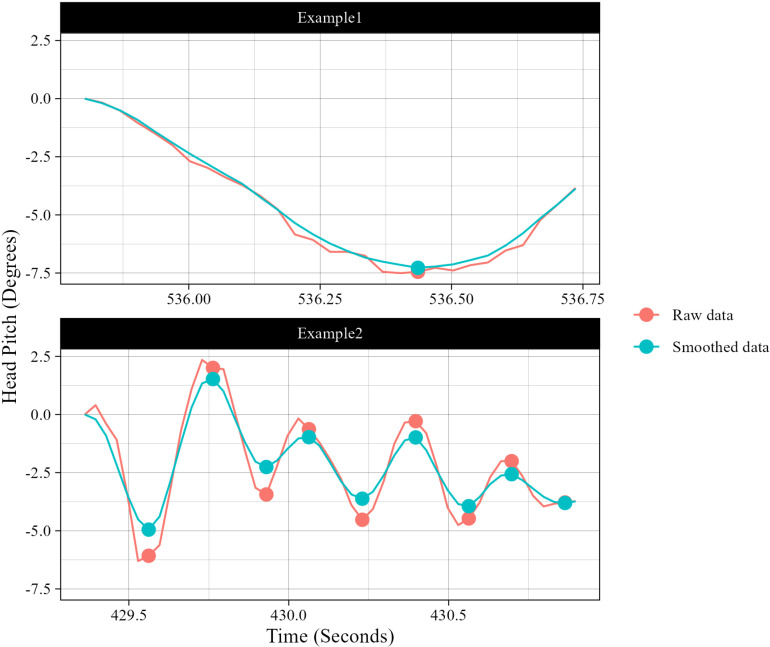
Example of inflection points estimated with a window size of 5.

Next, in order to calculate the magnitude, it is necessary to segment the nods into cycles. Furthermore, to segment them into cycles, they must first be divided into half-cycles, which requires identifying the inflection points of the movement. We observed minor noises in the obtained pitch values due to estimation errors. These noises posed a problem in estimating inflection points, as described later. To address this issue, we applied smoothing using a moving average window. To determine the optimal window size, we experimentally annotated all 68 nods observed in session 2 of Group 1’s conversation, marking the upward and downward movement intervals manually. Each upward and downward interval corresponds to a half-cycle, which allowed us to determine how many half-cycles each nod consists of. Next, we estimated the inflection points from the smoothed data for each window size. We calculated the difference between consecutive data points in the smoothed data and identified points where the polarity changed compared to the previous data point as inflection points. Since the inflection points lie at the connection points between half-cycles, the number of half-cycles can be estimated as the number of inflection points plus 1. We calculated the mean absolute error (MAE) between the estimated number of half-cycles and the manually annotated half-cycles, using the latter as the ground truth. The window size that resulted in the smallest MAE was selected as the optimal size. The MAEs for each window size are shown in Table 1. From the table, the MAE for a window size of 5 was the smallest at 0.82, which means that, on average, the error between the ground truth and the estimated values was less than 1. Therefore, we opted to use a window size of 5 for this study. Seven nods were extremely short, lasting only one frame, making it impossible to calculate the difference with the next frame; these were excluded, resulting in a total of 9,223 nods. Examples of estimated inflection points are shown in Fig 3. The vertical axis represents head pitch in degrees, while the horizontal axis represents time in seconds. For clarity, the pitch values have been adjusted so that the initial position of each nod is set to zero. The red line represents the raw data, while the blue line shows the smoothed data using a moving average window of size 5. The dots indicate the estimated inflection points. The nod in the upper panel has a length of 1 and a duration of 0.94 seconds, while the nod in the lower panel has a length of 5 and a duration of 1.53 seconds.

**Table 1:** MAEs of each window size.

Window size	MAE
0 (no smoothing)	3.22
3	1
5	0.82
7	0.84
9	1.29

**Fig 4:**
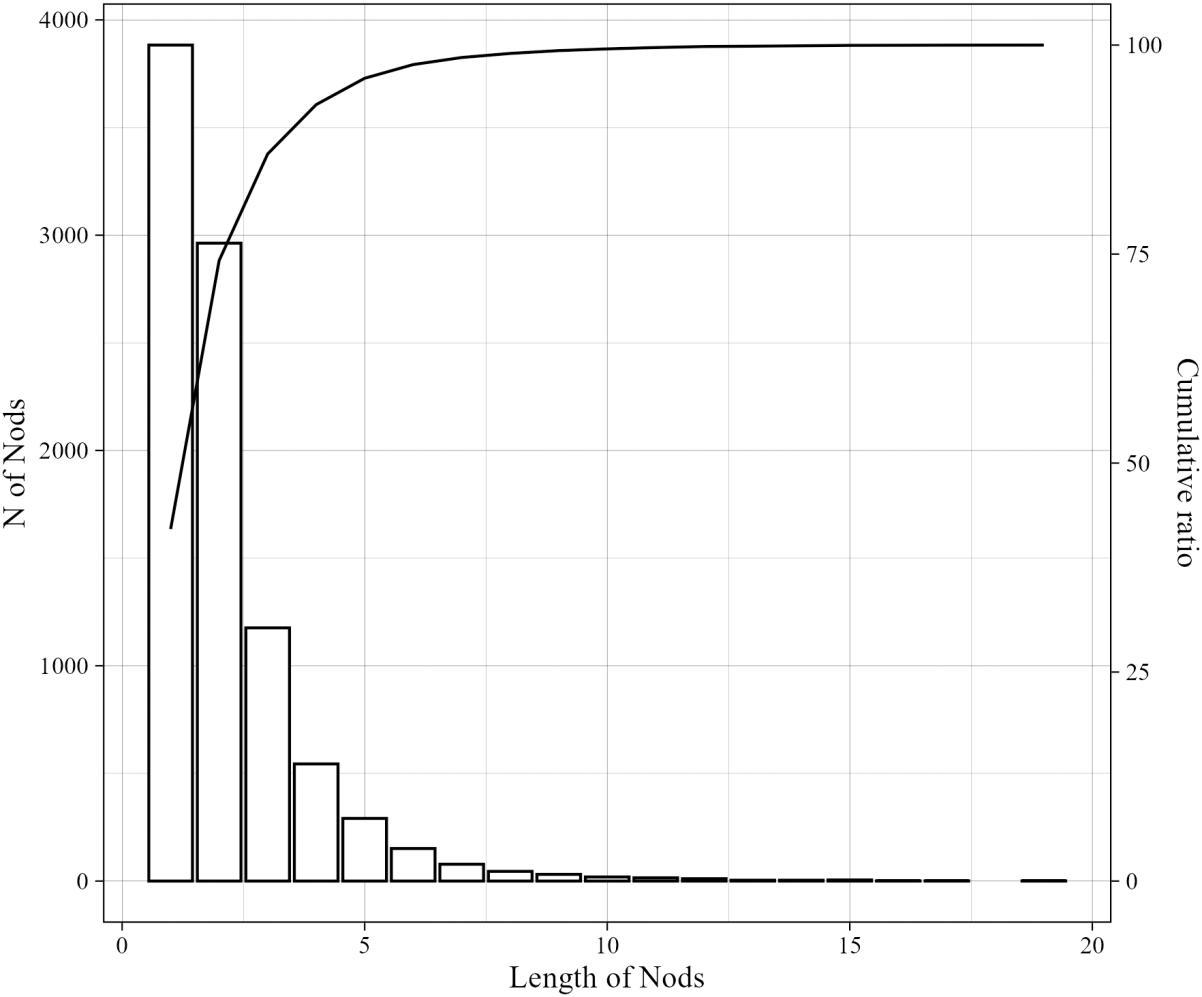
Distribution of nod lengths.

Based on the identified half-cycles, we recognized cycles and calculated the length of nods and the magnitude of each cycle. Fig 4 presents the distribution of nod lengths. The bars represent the number of nods for each length, while the line indicates the cumulative ratio. The most frequent length is one (single nods), accounting for 42% of the total, and the maximum length of repetitive nods is nineteen. Nods with a length of 1–5 account for 8,857 instances, making up more than 95% of all nods. In contrast, nods with a length of 6 or more total only 366 instances. Based on this observation, we decided to primarily focus on nods with lengths ranging from one to five for the subsequent analysis.

## Analysis

### Observation

**Fig 5:**
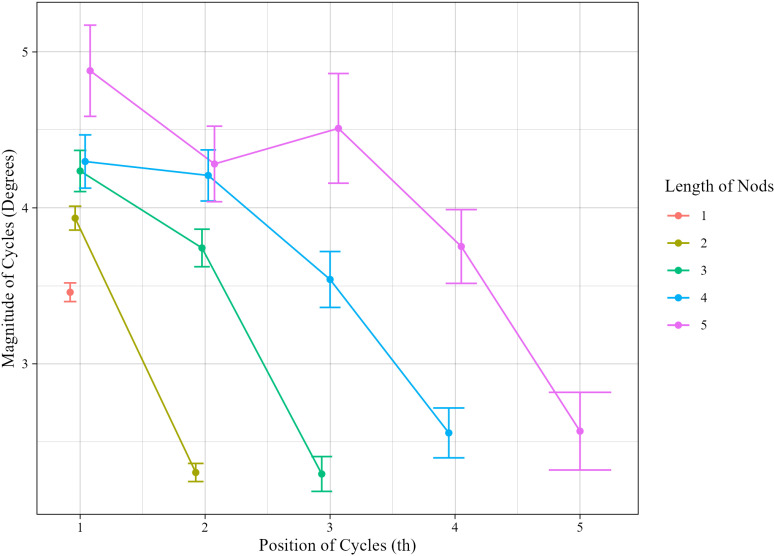
Average magnitude with respect to the length of nods and position of cycles.

Fig 5 shows the average magnitude of nods with lengths ranging from one to five, in relation to length of nods and position of cycles. We excluded outliers from the data. Firstly, our data contained two nods with extremely large cycles, having a magnitude greater than 90 degrees. This is likely due to misestimations in the head pose estimation. Secondly, the magnitude should theoretically be greater than 0. However, our data included 52 nods with cycles that had a magnitude of 0. This is thought to be because the actual magnitude was so small that it could not be detected by the head pose estimation, or due to segmentation errors, i.e., the data included periods where the movement had already ceased. After excluding these outliers, we used 8,803 nods and 16,843 cycles hereinafter. The error bars represent the standard error of the entire data. However, it should be noted that each cycle is derived from the same speaker or the same nod and is therefore not inherently independent. From the figure, the following observations can be made.

Firstly, the magnitude of the first cycles appears to increase proportionally with length. However, the difference of the magnitude of the first cycles between consecutive lengths does not seem to be equidistant but rather gets gradually smaller as the length gets long except for nods of length 5, which have a large standard error. This suggests that the increase would be monotonic and concave rather than linear with respect to the length.

Secondly, in contrast to the first cycles, the magnitude of the last cycles of repetitive nods is almost the same regardless of the length.

Thirdly, for all repetitive nods, there is a downward trend where the first cycles have the greatest magnitude, and the last cycles have the smallest magnitude. This indicates a potential decreasing effect with respect to position. As an exception, in nods of length 5, the magnitude appears to increase from the second to the third cycle. However, since their error bars greatly overlap, this trend may be by chance. Since the magnitude of the first cycles varies, it is unclear whether the slope is constant or varies with length and what the nature of that change would be—whether it is linear, a concave curve, or a convex curve.

Lastly, the reduction in the magnitude of the last cycles is particularly larger compared to the prior positions. This suggests the presence of an additional reduction effect specifically acting on the last cycles in a sequence. It is also unclear whether this effect is constant or varies with length and what the nature of that change would be.

The questions derived from the above observations are summarized as follows. Q1 and Q2 pertain to the magnitude of the first and last cycles, respectively. Q3 concerns the change in the magnitude of cycles with position, while Q4 addresses the reduction effect that affects only the final cycles.

Q1-1: Is the magnitude of the first cycles constant or variable with length?

Q1-2: If the magnitude of the first cycles is variable with length, what would be the nature of that change?

Q2-1: Is the magnitude of the last cycles constant or variable with length?

Q2-2: If the magnitude of the last cycles is variable with length, what would be the nature of that change?

Q3-1: Does the magnitude of cycles decrease with position?

Q3-2: If there is a decreasing trend, is it constant or variable with length?

Q3-3: If the decreasing trend is variable with length, what would be the nature of that change?

Q4-1: Is there an additional reduction effect at the last cycles?

Q4-2: If there is an additional reduction effect, is it constant or variable with length?

Q4-3: If the additional reduction effect is variable with length, what would be the nature of that change?

### Modeling

To clarify the questions derived from the observations, we built statistical models that predict the magnitude at each position and for each length.


$$y_{i}\;\sim \;Gamma(k,\;\mu_{i}/\;k)$$

$$log(\mu_{i})\;=\;fixed({position}_{i},\;{length}_{i})\;+\;\alpha_{n}\;+\;\alpha_{p}$$

$$\alpha_{n}\;\sim \;Normal(0,\;\sigma_{n})$$

$$\alpha_{p}\;\sim \;Normal(0,\;\sigma_{p})$$

We employ a generalized linear mixed-effects model. Here, the dependent variable *y* represents the magnitude of each cycle. Since it is greater than 0, it is assumed to follow a Gamma distribution. Gamma distribution has two parameters, the shape parameter *k* and the scale parameter *θ* = *μ*/ *k*. The mean of the Gamma distribution, calculated as *μ = kθ*, is linked to the linear predictor through a link function, here assumed to be a log function. The linear predictor consists of two parts: fixed-effects part and random-effects part. The fixed-effects part will be described below. The random-effects part represents variations due to individual participants and instances. The magnitude value of each cycle may be taken from the same nod or from nods produced by the same participant. Thus, these values are not independent of each other, which motivate us to use random effects for individual instances and individual participants. As random effects, we used two parameters. *α*_*n*_ represents the random intercept for the nod that the cycle in question belongs to. This is based on the assumption that because the magnitude of cycles in a nod is affected by factors such as the intensity of that nod as a response, the cycles belonging to the same nod share the common baseline, i.e., intercept. Similarly, *α*_*p*_ represents the random intercept for participant. This assumes that the magnitude varies among participants, with some participants consistently having larger magnitudes and others consistently having smaller magnitudes.

For the fixed effects, we compared the following eight models.

Model1: *fixed*(*position*, *length*) = *a* + *b* (*position* – 1) + *c final*

Model2: *fixed*(*position*, *length*) = *a* + *b*_*length*_ (*position* – 1) + *c final*

Model3: *fixed*(*position*, *length*) = *a* + *b* (*position* – 1) + *c*_*length*_ *final*

Model4: *fixed*(*position*, *length*) = *a* + *b*_*length*_ (*position* – 1) + *c*_*length*_ *final*

Model5: *fixed*(*position*, *length*) = *a*_*length*_ + *b* (*position* – 1) + *c final*

Model6: *fixed*(*position*, *length*) = *a*_*length*_ + *b*_*length*_ (*position* – 1) + *c final*

Model7: *fixed*(*position*, *length*) = *a*_*length*_ + *b* (*position* – 1) + *c*_*length*_ *final*

Model8: *fixed*(*position*, *length*) = *a*_*length*_ + *b*_*length*_ (*position* – 1) + *c*_*length*_ *final*

The fixed-effects part of the linear predictor, in general, involves three parameters: *a*, *b*, and *c*. *a* represents the magnitude of the first cycles. *b* is the coefficient for the trend according to position, and it takes effect when the position is greater than 1, as indicated by (*position* - 1). *c* is the coefficient for the additional effect at the last cycles, with *final* being a binary variable that is 1 when the cycle is the final one, i.e., *position = length*, and 0 otherwise. In addition, there are two cases for each parameter; those without subscripts are constant across lengths, while those with the length subscript are re-parameterized with respect to length as follows:


$$a_{length}=\alpha_{a}+\beta_{a}\;{\left(length\;–\;1\right)}^{\gamma a}$$

$$b_{length}=\alpha_{b}+\beta_{b}\;{\left(length\;–\;2\right)}^{\gamma b}$$

$$c_{length}=\alpha_{c}+\beta_{c}\;{\left(length\;–\;2\right)}^{\gamma c}$$

When these parameters are re-parameterized, we assume that they can be modeled as a power function of length with some exponent, involving three new parameters: *α*, *β*, and *γ*. *α* is the intercept at the minimum possible value of length, i.e., *length *= 1 for *a*_*length*_ and *length* = 2 for *b*_*length*_ and *c*_*length*_. *β* determines the direction and magnitude of a change in the parameter value along length. *γ* represents the exponent of the power function. For example, if *β* has a positive value, the function is concave when *γ* is between 0 and 1, linear at *γ = *1, and convex for *γ* values greater than 1. Because for negative *γ* the sign and direction of *β* are reversed, *γ* is constrained to be non-negative.

The parameters *a*, *b*, and *c* correspond to Q1, Q3, and Q4, respectively. If these parameters are constant with respect to length, then models using constants will likely be adopted. On the other hand, if the parameters vary, re-parameterized models will be employed. Q2 will be addressed by estimating the magnitude of the last cycles using estimated these parameters.

Note that in all models, when the length is 1, the position-dependent change *b* and the effect on the last cycles *c* are not considered, and, thus, a simpler model, *fixed*(*position*, *length*) = *a* or *a*_*length*_, is used. This assumes that the additional effect at the last cycles, *c*, only works on the repetitive nods.

The parameters were estimated utilizing Markov Chain Monte Carlo (MCMC) method, employing the statistical analysis language R (version 4.4.2) and the rstan package (version 2.32.6) as an interface to the probabilistic programming language Stan within the R. We adopted non-informative prior distributions of a zero-centered normal distribution with a standard deviation of 10 for *a*, *b*, *c*, *α*, *β* and *γ*, and a half Cauchy distribution with 0 as the position parameter and 5 as the scale parameter for the parameter *k* and the standard deviation of random effects, *σ*_*n*_ and *σ*_*p*_. The burn-in period was always set to 1000, and the total number of iterations and the thin were set to 4000 and 3, respectively, except for Model3 and 8. For convergence, they were set to 5000 and 4, respectively for Model3 and 8. The rstan’s default number of chains is 4, but for obtaining robust results, this study utilized 8 chains. Thus, we finally obtained 8000 samples for each model. Convergence was assessed through multiple methods including trace plots, Gelman-Rubin diagnostic, and bulk-ESS, and we confirmed that all Rhat values for the parameters remained below 1.05.

For model comparison, the loo_comparison function from the loo package (version 2.8.0) was used. The loo_comparison function compares models based on the expected log pointwise predictive density (elpd) calculated using leave-one-out cross-validation (LOO). It outputs the elpd for each model (elpd_loo), the difference from the model with the highest elpd (elpd_diff), and the standard error of the difference (se_diff). When the significance level is set to 95%, if the range of elpd_diff ± 1.96 * se_diff includes zero, there is no significant difference between that model and the optimal model. According to the principle of Occam’s razor, if there was no significant difference between models, the simpler model with fewer parameters was selected.

## Results

Table 2 shows the results of the parameter estimation and model comparison. The values in the parameter rows represent the median of the posterior distribution for each parameter, with * indicating significance at the 95% level. Additionally, * in the elpd_diff row indicates a significant difference from the model with the highest elpd_loo.

**Table 2:** Results of parameter estimation and comparison.

	Model1	Model2	Model3	Model4	Model5	Model6	Model7	Model8
*a*	1.171*	1.177*	1.176*	1.175*				
*α*_*a*_					1.087*	1.084*	1.086*	1.086*
*β*_*a*_					0.093*	0.118*	0.110*	0.115*
*γ*_*a*_					0.865*	0.673*	0.746*	0.711*
*b*	-0.045*		-0.063*		-0.098*		-0.120*	
*α*_*b*_		-0.150*		-0.088		-0.237*		-0.159
*β*_*b*_		0.056*		0.012		0.078*		0.018
*γ*_*b*_		0.558*		0.282*		0.557*		0.367*
*c*	-0.561*	-0.480*			-0.509*	-0.409*		
*α*_*c*_			-0.570*	-0.548			-0.525*	-0.491
*β*_*c*_			0.039*	0.036			0.050*	0.046
*γ*_*c*_			1.210*	0.592*			1.278*	0.699*
*σ*_*n*_	0.492*	0.493*	0.493*	0.493*	0.492*	0.494*	0.493*	0.493*
*σ*_*p*_	0.301*	0.301*	0.300*	0.302*	0.297*	0.297*	0.297*	0.299*
*k*	1.962*	1.965*	1.965*	1.965*	1.976*	1.980*	1.980*	1.981*
elpd_loo	-34298.3	-34262.8	-34276.7	-34277.7	-34243.1	-34232.8	-34235.8	-34238.6
elpd_diff	-65.5*	-30.0*	-43.9*	-44.9*	-10.3	0	-3.0	-5.8
se_diff	9.6	9.1	8.8	8.9	6.2	0	4.5	5.4

First, looking at the elpd_loo values, there is a substantial difference between Model1–4, which use a constant *a*, and Model5–8, which use a version of *a* re-parameterized depending on length. Among Model5–8, Model6, which has the re-parameterized *a* and *b*, has the highest elpd_loo, but no significant differences are observed between Model6 and the other models. Therefore, we selected Model5 as the best model, which has the fewest parameters among them.

Next, let us focus on the selected Model5. This model employs variable *a* and constant *b* and *c*. First, looking at the parameter related to *a*, which represents the magnitude of the first cycles, all of the parameters, *α*_*a*_, *β*_*a*_, and *γ*_*a*_, are significant. *β*_*a*_ was estimated to be positive, 0.093, which indicates that the magnitude of the first cycles increases significantly with the length of repetitive nods. Therefore, the observation that the magnitude of the first cycles increases with length has been confirmed. Additionally, *γ*_*a*_ was estimated to be 0.865, which was between 0 and 1, indicating a monotonically increasing concave pattern. This means that the difference of the magnitude of the first cycles, *a*_*length*_, gets gradually smaller as the length gets long. Therefore, the magnitude of the first cycles is a function of length (satisfying Q1-1), and the change follows a monotonically increasing concave pattern (satisfying Q1-2).

Examining *b*, which represents changes due to position, it was negative and significant, -0.098, indicating a significant decreasing trend with position. Given that *b* is constant in the best model, this slope remains almost constant regardless of length. Therefore, the magnitude of cycles decreases with position (satisfying Q3-1), and this trend is constant across lengths (satisfying Q3-2).

Looking at *c*, which represents the additional effect on the last cycles, it was negative and significant, -0.509. This result implies a significant reduction effect that affects specifically the last cycles. Since *c* was also a constant in the best model, this effect is considered to be constant regardless of length. Therefore, there is an additional reduction effect applying to the last cycles (satisfying Q4-1), and this effect is constant across lengths (satisfying Q4-2).

**Fig 6:**
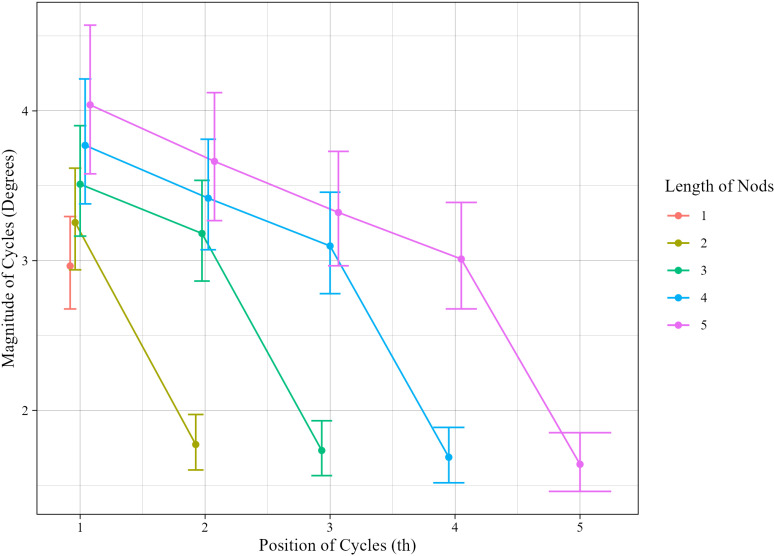
Posterior distribution of fixed effects.

Fig 6 shows a posterior distribution of fixed effects in Model5. The error bars represent the 95% credible intervals. As was stated above, the magnitude of the first cycles gets greater as the length of the nod gets longer, indicating an *anticipatory* effect that the length of the nod to be generated is anticipated from the beginning. Then, the magnitude is reduced as a linear function of position with a constant declination trend irrelevant of length. The last cycles of repetitive nods reveal an additional reduction effect; large overlapping intervals for the magnitude of the last cycles among the estimated posterior distributions for different lengths suggest that there was no significant difference among them. Therefore, the magnitude of the last cycles seems to be constant irrelevant of lengths (satisfying Q2-1).

The above models represent the magnitude as changing linearly with respect to position. However, looking at Fig 6, one might argue that this linearity oversimplifies the trend. In fact, Fig 5 suggests that the observed values exhibit a curved pattern, specifically resembling a quadratic decay. Therefore, we performed an additional analysis, comparing Model5 and quadratically decaying models.

In Model5, the part representing the decay due to position is *b* (*position* – 1). Assuming the decay with position is quadratic, the model would be as follows.

Model9: *fixed*(*position*, *length*) = *a*_*length*_ + *b* (*position*^2–1) + *c final*

Table 3 shows the results of comparison between Model5 and Model9. For the estimation of Model 9, the burn-in period was set to 1,000, the total number of iterations to 5,000, and the thin to 4. As shown in the table, Model5 has a significantly higher elpd than Model9.

**Table3:** Results of comparison between Model5 and Model9.

	Model5	Model9
elpd_loo	-34243.1	-34267.4
elpd_diff	0	-24.3*
se_diff	0	5.2

Additionally, assuming that the manner of decay changes with the length in the same way as the first cycles, we also examined more complex Model10, which can be expressed as follows.

Model10: *fixed*(*position*, *length*) = *a*_*length*_ + *b*_*length*_ (*position*^2–1) + *c final*

Table 4 shows the results of comparison between Model5 and Model10. Model 10 was estimated using the same settings as Model 9. The comparison of elpd results shows that Model10 has the highest elpd. However, there is no significant difference between Model10 and Model5. Nevertheless, Model10 has more parameters than Model5, and following Occam’s Razor, Model5, which is simpler with a similar elpd, is chosen. From these results, it can be said that our model generalizes the data appropriately without compromising elpd.

**Table4:** Results of comparison between Model5 and Model10.

	Model5	Model10
elpd_loo	-34243.1	-34231.9
elpd_diff	-11.2	0
se_diff	6.4	0

Finally, the answers to the questions obtained from the observations can be summarized as follows.

Q1-1: The magnitude of the first cycles significantly increases with length.

Q1-2: The change follows a monotonically increasing concave pattern.

Q2-1: The magnitude of the last cycles is constant with length.

Q2-2: No answer since the premise is not fulfilled (the answer to Q2-1 is constant).

Q3-1: The magnitude of cycles significantly decreases with position.

Q3-2: The change is constant with length.

Q3-3: No answer since the premise is not fulfilled (the answer to Q3-2 is constant).

Q4-1: There is a significant reduction effect at the last cycles.

Q4-2: The change is constant with length.

Q4-3: No answer since the premise is not fulfilled (the answer to Q4-2 is constant).

## Discussion

The analysis revealed that the magnitude of the first cycles increases as the length of the nod increases, following a monotonically increasing concave pattern. Additionally, the magnitude of cycles decreases with a constant slope from that point, eventually reaching a stable lowest magnitude regardless of length. It was also found that the magnitude of the last cycles is significantly smaller than is expected by the decreasing trend.

Interestingly, these patterns closely resemble tendencies observed in phonological structures. Phonological studies have shown that the pitch of the voice tends to decrease from the beginning to the end of an utterance, a phenomenon known as *declination* [28]. Declination is a physiological phenomenon associated with a decrease in breath [29], making it a universal phenomenon observed in a wide range of languages, including tone languages such as Mandarin [28,30–34]. Our finding that the magnitude of cycles decreases with position, a result consistent with Harder et al.‘s [4] observations, is closely resembles this phonological fact. However, what is a source of a similar decreasing effect in nods? It is likely that, in the case of nods, an initial intense stimulus—similar to an impulse input to a spring-mass-damper system—propels the movement, and the movement continues without additional force by utilizing that initial energy. As time progresses, the energy diminishes, resulting in a decrease in the cycle magnitude. The fact that the pattern of reduction is constant with respect to the length would also support this idea.

A similar phenomenon to the increasing magnitude of the first cycles with longer nods is observed in speech, where the pitch at the beginning of a longer utterance tends to be higher, a phenomenon referred to as *anticipatory rising* [30,35,36]. In speech, it is attributed to the need for greater exhalation to sustain a long utterance. If the decreasing trend in the magnitude of cycles during nods is due to the reason mentioned above, it can be inferred that longer nods require more intense initial force, leading to this observed pattern. Moreover, anticipatory rising is interpreted as evidence that the speaker has a long-term plan for the utterance at the start of speaking [35]. Similarly, this trend in nods could be evidence that the person nodding has a prior plan at the outset regarding the approximate number of nods to produce. Interestingly, this initial effect, as well as the constant reduction trend, might also enable co-participants to predict how long the nod will continue, only based on the initial part of the nod, which serves as an important interactional resource. According to Bauer et al. [15], in German sign language, recipient’s affirmative nods are faster and have larger amplitudes compared to nods used for feedback. Additionally, since most affirmative nods are used just before the recipient takes the turn, they suggest that these characteristics might function as signals indicating that the recipient will take the turn. In light of the findings from this study, the characteristics of the recipient’s nod might not only serve as a cue not only for whether to take a turn but also for when to take it.

Turning to the last cycles, a similar reduction effect on the significantly smaller last cycles is observed in speech, where the pitch markedly decreases at the end of an utterance, a phenomenon known as *final lowering* [32–34,37,38]. This phenomenon is considered a marker indicating the end of the utterance, which might be also functioning in nods for marking the end of the repetitive nod. Additionally, the final pitch in the utterance tends to stabilize at a certain level, regardless of the utterance length [33,35]. This is also parallel to our finding that the last cycles’ magnitude remains consistent regardless of the nod length, Thus, the final elements of both nods and speech might be marked with two characteristics: a markedly smaller value than prior positions and consistently stable level, regardless of length.

The results of this study have made it clear that nods are not merely a random up-and-down motion but possesses a distinct structure. Furthermore, this structure shares many commonalities with the phonological structure of speech. This suggests that the structure of human repetitive actions might be universal regardless of modality.

## Conclusion

In this study, we focused on the structure of nods, which has often been considered obvious and overlooked, and demonstrated that nods are not just a random up-and-down movement of the head but rather possesses a distinct structure. First, prior to analysis, we proposed systematic conceptualization related nods as a head movement, and defined terms such as cycle, half-cycle, magnitude, length, and position. These terms will likely serve as useful tools for describing and analyzing nod movement in future research. The statistical analysis revealed that the motion of nods has a structure in which the magnitude of cycles decreases with position, the magnitude of the first cycles increases with the length of the nod, and the magnitude of the last cycles is markedly reduced. Furthermore, the similarity between these structures in nods and phonological structures in speech suggests an intriguing possibility that they may represent a universal structure in human repetitive actions.

The findings of this study offer several implications. First, the observed similarities between nods and speech suggest that there might be many other features and structures that are shared between speech and body movements beyond what was found in this study. Second, the model developed in this research could be utilized by embodied conversational agents when generating more natural nod gestures.

One limitation of this study is that the data primarily consists of Japanese speakers in their 20s. While there is no strong evidence suggesting that the structure of nods varies with age, the frequency and functions of nods are known to differ across cultures [5,6], implying the possibility of cultural influences. Even though a part of our findings is consistent with Harder et al.‘s [2] observations, it still remains unclear whether the structures observed in this study are universal across different cultures. As a direction for future research, we will compare the structure observed in this study with the structures in other cultures and analyze its relationship with the phonological structure of the respective language. Furthermore, unlike previous studies, the data in this study are derived from three-party conversations. It is unclear whether the number of participants in a conversation affects the structure of nods, but this could be also an interesting topic for future research.

Nods are a gesture frequently observed in daily conversations. At first glance, they seem like a simple movement, but even such seemingly simple movements possess some structure influenced by factors such as bodily structure and cognitive abilities, and this structure is likely to be used as a resource in interaction. We hope that the results of this study will provide new perspectives on other gestures than nods; because the structure of nods is influenced by factors such as bodily structure and cognitive abilities, they may share similar characteristics with structures in other modalities. This reminds us that research in seemingly disparate fields, such as phonology and gestures, is fundamentally the study of the same objective, i.e., human beings.
